# Ischemic Heart Disease and Vascular Risk Factors Are Associated With Accelerated Brain Aging

**DOI:** 10.1016/j.jcmg.2023.01.016

**Published:** 2023-07

**Authors:** Elisa Rauseo, Ahmed Salih, Zahra Raisi-Estabragh, Nay Aung, Neha Khanderia, Gregory G. Slabaugh, Charles R. Marshall, Stefan Neubauer, Petia Radeva, Ilaria Boscolo Galazzo, Gloria Menegaz, Steffen E. Petersen

**Affiliations:** a William Harvey Research Institute, National Institute for Health Research (NIHR) Barts Biomedical Research Centre, Queen Mary University London, Charterhouse Square, London, United Kingdom; b Barts Heart Centre, St Bartholomew’s Hospital, Barts Health National Health Service (NHS) Trust, West Smithfield, London, United Kingdom; c Department of Computer Science, University of Verona, Verona, Italy; d Imperial College Healthcare NHS Trust, London, United Kingdom; e School of Electronic Engineering and Computer Science, Queen Mary University of London, United Kingdom; f Alan Turing Institute, London, United Kingdom; g Digital Environment Research Institute, Queen Mary University of London, London, United Kingdom; h Preventive Neurology Unit, Wolfson Institute of Population Health, Charterhouse Square, London, United Kingdom; i Neurology Department, Royal London Hospital, Barts Health NHS Trust, London, United Kingdom; j Division of Cardiovascular Medicine, Radcliffe Department of Medicine, University of Oxford, Oxford NIHR Biomedical Research Centre, Oxford University Hospitals NHS Foundation Trust, Oxford, United Kingdom; k Department of Mathematics and Computer Science, University of Barcelona, Barcelona, Spain; l Health Data Research UK, London, United Kingdom

**Keywords:** brain aging, brain health, cognitive decline, ischemic heart disease, vascular risk factors

## Abstract

**Background:**

Ischemic heart disease (IHD) has been linked with poor brain outcomes. The brain magnetic resonance imaging–derived difference between predicted brain age and actual chronological age (brain-age delta in years, positive for accelerated brain aging) may serve as an effective means of communicating brain health to patients to promote healthier lifestyles.

**Objectives:**

The authors investigated the impact of prevalent IHD on brain aging, potential underlying mechanisms, and its relationship with dementia risk, vascular risk factors, cardiovascular structure, and function.

**Methods:**

Brain age was estimated in subjects with prevalent IHD (n = 1,341) using a Bayesian ridge regression model with 25 structural (volumetric) brain magnetic resonance imaging features and built using UK Biobank participants with no prevalent IHD (n = 35,237).

**Results:**

Prevalent IHD was linked to significantly accelerated brain aging (*P <* 0.001) that was not fully mediated by microvascular injury. Brain aging (positive brain-age delta) was associated with increased risk of dementia (OR: 1.13 [95% CI: 1.04-1.22]; *P =* 0.002), vascular risk factors (such as diabetes), and high adiposity. In the absence of IHD, brain aging was also associated with cardiovascular structural and functional changes typically observed in aging hearts. However, such alterations were not linked with risk of dementia.

**Conclusions:**

Prevalent IHD and coexisting vascular risk factors are associated with accelerated brain aging and risk of dementia. Positive brain-age delta representing accelerated brain aging may serve as an effective communication tool to show the impact of modifiable risk factors and disease supporting preventative strategies.

Although the risk of cognitive decline is primarily driven by age, vascular risk factors and coexisting diseases may augment the risk of age-related brain deterioration.^1^^,^^2^ Ischemic heart disease (IHD) has been associated with poor brain health independently of the effect of aging itself.^3^^,^^4^ Several pathways involved in heart-brain crosstalk have been proposed, albeit the precise pathophysiological mechanisms by which IHD may cause cognitive deterioration remain to be elucidated.^5^ Cardiac ischemia has been linked with macro- and microstructural brain abnormalities in both white and grey matter even before the onset of clinical symptoms of dementia.^6^^,^^7^ Detecting and quantifying subtle deviations from age-related brain changes may enable identification of subjects at greater risk for developing cognitive decline to whom preventive strategies and targeted treatments should be directed.

Recent neuroimaging studies have introduced modelling approaches to estimate individual brain age based on brain magnetic resonance imaging (MRI) features. The difference between estimated brain age and actual age (delta) reflects deviations from normal aging and may serve as a biomarker for cognitive deterioration.^8^ An older-appearing brain indicating accelerated brain aging (positive delta) has been linked with certain neurological conditions and lifestyle factors in cognitively healthy cohorts.^9^^,^^10^

Brain age may represent an effective means of communicating the risk of brain deterioration to patients, just as vascular age can be used to express cardiovascular disease risk.^11^ It could increase risk awareness and likely promote healthier lifestyles and preventive actions.^12^

We have investigated the impact of prevalent IHD on brain aging and the risk of dementia, possible mechanisms underlying the association, and the role of vascular risk factors, cardiovascular structure, and function using UKB (United Kingdom Biobank) data. No previous studies have used this approach to evaluate brain health in large IHD cohorts without pre-existing neurological conditions. We hypothesize that IHD may be associated with accelerated brain aging, reflecting structural brain changes predisposing to cognitive decline or dementia. The estimated brain age can thus be used to express the individual risk level and motivate IHD patients to improve their health behaviors.

## Methods

### The UKB data set

The UKB is a large prospective population study following the health and wellbeing of more than half a million participants 40-69 years of age who were recruited from across the United Kingdom. The protocol is publicly accessible, and data are made available for researchers through an application process.^13^ Participants’ characteristics, including sociodemographics, lifestyle, environmental factors, medical history, genetics, and physical measures were collected at the visits. Since 2015, more than 48,000 participants underwent imaging studies, including brain and cardiovascular magnetic resonance (CMR) imaging.

### Ethics

This study complies with the Declaration of Helsinki. It is covered by the ethical approval for UKB studies from the National Health Service (NHS) National Research Ethics Service on June 17, 2011 (Reference 11/NW/0382), and extended on June 18, 2021 (Reference 21/NW/0157). Written informed consent was obtained from all participants.

### Study populations

**Figure 1:**
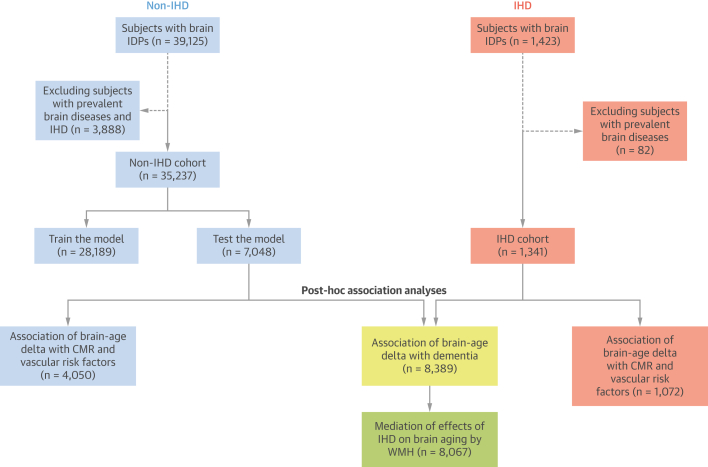
Study Population Selection Created using BioRender.com. CMR = cardiac magnetic resonance; IDP = imaging-derived phenotype; IHD = ischemic heart disease; WMH = white matter hyperintensity.

Participants with brain imaging–derived phenotypes (IDPs) available and without any history of mental health, neurological disorders, or dementia that could directly affect cognitive function were selected for our analysis (n = 36,578).^14^ Two distinct subsets were then defined (Figure 1). The prevalent IHD group comprised 1,341 subjects with angina, previous myocardial infarction, or any manifestation of IHD not resulting in infarction (Supplemental Table 1). The non-IHD (control) group was composed of the remaining subjects with no history of IHD at the time of scanning (n = 35,237). This group was further split into training and test sets to define the brain aging model. For the post hoc association analyses (performed on the IHD and non-IHD test sets), we selected only subjects with available values for the exposure variable of interest.

### MRI data acquisition

Brain MRI data were acquired using a 3.0-T Siemens scanner (Skyra, VD13A SP4, Siemens Healthcare) in accordance with a publicly accessible protocol.^15^ In brief, imaging acquisition included 6 different sequences, covering structural, diffusion, and functional imaging for a 35-minute scan time per participant. MPRAGE sequence with 1-mm isotropic resolution was used to acquire T1-weighted data, while fluid-attenuated inversion recovery contrast was used for T2-weighted scans (1.05 × 1 × 1-mm resolution).

CMR images were acquired using 1.5-T scanners (MAGNETOM Aera, Syngo Platform VD13A, Siemens Healthcare) according to a predefined acquisition protocol.^16^ In brief, the cardiac assessment includes a combination of long-axis cines and a complete short-axis stack covering both the left ventricle (LV) and right ventricle (RV) acquired using balanced steady-state free precession sequences.

### Brain MRI feature extraction

Brain IDPs were extracted from brain MRI and accessible through the UKB showcase (Supplemental Table 2). We selected 25 volumetric features extracted from structural MRIs as they were identified among the top informative predictors to model brain age.^17^^,^^18^ The total volume of white matter hyperintensities (WMHs) (not used to model brain age) was instead used for the post hoc mediation analysis. A detailed description of processing data is provided in the Supplemental Methods.

### CMR parameters

Conventional measures of LV and RV structure and function were extracted using an automated pipeline with inbuilt quality control, previously developed, and validated in a large sample of the UKB.^19^ Specifically, we analyzed the following parameters: LV and RV volumes in end-diastole and end-systole; LV and RV stroke volumes; LV mass; and left ventricular ejection fraction.

Additional cardiac indices able to capture changes in myocardial function in relation to structural chamber remodeling were analyzed, including LV mass-to-volume ratio, and LV global function index. Mass-to-volume ratio was calculated as LV mass divided by LV end-diastolic volume. LV global function index is a validated measure of LV cardiac performance that integrates structural components of adverse myocardial remodeling into LV function assessment for which the formula is described elsewhere.^20^

Aortic distensibility and total arterial compliance (TAC) obtained at the imaging visit were considered as indices of arterial health. Aortic distensibility is a measure of local aortic compliance and lower values indicates poor vascular health.^21^ Aortic distensibility values at the proximal descending aorta were obtained from a previous analysis of a large subset of the UKB imaging studies using a fully automated image analysis pipeline embedded with purpose-designed quality control.^22^ TAC is an additional descriptor that can be used to estimate arterial function. Greater values, indicating reduced arterial load, have been associated with better cardiovascular outcomes.^23^ TAC was estimated using LV stroke volume divided by central pulse pressure.^24^

### Vascular risk factors

Vascular risk factors previously linked with brain health, including hypertension, diabetes, hypercholesterolemia, and (current) smoking were considered as exposures. Body mass index and waist-hip ratio, 2 biomedical indices of body adiposity, were also evaluated. We also assessed the role of socioeconomic deprivation, expressed as Townsend index, a measure of material deprivation relative to national averages. The clinical exposures were ascertained using selected UKB fields (Supplemental Table 3).

### Statistical analysis

A more detailed description of the statistical analysis is provided in the Supplemental Methods. All analyses were performed using Python 3.8.10 (Python Software Foundation) and Scikit-learn version 0.23.2. A 2-sided value of *P <* 0.05 (corrected for multiple tests) was considered statistically significant for all analyses.

Briefly, to assess whether IHD was associated with faster brain aging, whether it, in turn, was linked with dementia risk, and to investigate potential mechanistic links and risk factors involved in the association, we used a staged approach, which can be summarized as follows (Supplemental Figure 1).

#### Brain age estimation

Brain age was estimated using Bayesian Ridge regression model as it was shown to provide competitive performance.^25^ In the model, 25 brain IDPs were the independent variables while the chronological age was the dependent variable. The actual age was demeaned (shifted to have zero mean) before fitting it into the model to have a centered version of the outcome.^8^ Sex, education level, height, and volumetric scaling from T1 head image to standard space were used as confounds as they can significantly affect the outcome. The confounds were regressed from the features using a linear regression model before modeling brain age. The model performance was assessed using the mean absolute error (MAE) and the coefficient of determination (R^2^). MAE in brain age studies is interpreted as the deviation between predicted brain age and the chronological age expressed in years, with higher values indicating older appearing brains. R^2^ represents the proportion of variance in the predicted brain age explained by the used features in the model.

The brain aging model was first built based on participants with non-IHD by splitting the data into 2 subsets (training set, 80%; testing set, 20%). After removing the age-dependency bias (as described in the Supplemental Methods), we calculated brain-age delta by subtracting the chronological age from the predicted brain age in the test data sets, with positive values (expressed in years) indicating accelerated brain aging. Pearson correlation was also calculated between actual age and brain-age delta, before and after bias correction.

Next, brain age was estimated on IHD subjects using the previously trained model to assess the deviation of the brain-age delta from the reference (non-IHD) population. IHD and non-IHD groups were thus compared in terms of model performance and difference in mean brain-age delta, the latter of which was considered as a measure of apparent brain aging.^8^

#### Brain age and risk of dementia

Logistic regression was used to analyze the relationship between brain-age delta and incident dementia (Supplemental Table 4). The model was adjusted for age, sex, and education level. As the number of incident events was low in comparison to nonevents, to account for imbalance data potentially affecting the model, we repeated the association analysis after using propensity score matching based on age and sex.

#### Mediation effects of WMH on IHD and brain age

To study potential mechanistic links, we performed a mediation analysis to test to which extent WMH, a marker of cerebrovascular injury, mediated the impacts of IHD on brain aging (indirect effect). The ordinary least squares regression was used to study the associations (described in terms of effect) between the variables (IHD, WMH as mediator, and brain-age delta). The model was adjusted for age, sex, and education.

#### Association of brain age with vascular risk factors and imaging parameters

A linear regression model in both IHD and non-IHD (test-set) groups was used to assess the role of vascular risk factors and imaging parameters in advancing brain age. The model was adjusted for the same confounds used for brain aging model plus age. In the analysis, we used unstandardized measures to reveal the effect (beta value) of changing 1 unit in the exposure on the brain-age delta. Specifically, changing the exposures may lead to increasing or decreasing in brain-age delta (estimated in years) based on the direction of the association (positive vs negative beta value).

As we found significant associations between certain imaging parameters and brain aging only in non-IHD subjects, we studied whether these metrics were linked with incident dementia in this group using logistic regression. The model was adjusted for age and sex.

## Results

### Baseline characteristics of the study populations

People with IHD were on average older than the controls (59.2 ± 6.3 years vs 54.6 ± 7.4 years; *P <* 0.05), albeit the age range was similar in the 2 cohorts (40 to 70 years) (Supplemental Figure 2). The diseased subjects were more likely to be males than those without the disease (71.4% vs 46.3%, respectively; *P <* 0.05) (Table 1), with a greater burden of risk factors and worse cardiovascular health indices (Table 2). IHD subjects had significantly lower volumes of most brain structures and increased volume of WMH than those without the disease (Supplemental Table 5).

**Table 1:** Demographic Characteristics of the Study Groups Used for Brain Age Prediction

	Non-IHD (n = 35,237)	IHD (n = 1,341)
Age, at the imaging visit, y	**54.6 ± 7.44**	**59.2 ± 6.3**
Male	**16,338 (46.3)**	**958 (71.4)**

Values are mean (± SD) when continuous or number (%) when categorical. **Bold** indicates differences between the 2 groups are statistically significant (*P* < 0.05).

IHD = ischemic heart disease.

**Table 2:** Baseline Characteristics of the IHD vs Non-IHD Groups

	Non-IHD (Test-Set)	IHD
Clinical characteristics		
Deprivation (Townsend score)	−2.5 (−3.9 to −0.7)	−2.6 (−3.9 to −0.5)
Current smoker	**251 (6.1)**	**86 (8.0)**
Diabetes	**128 (3.1)**	**88 (8.2)**
Hypertension	**193 (4.7)**	**125 (11.6)**
High cholesterol	**566 (13.9)**	**204 (19)**
Biomedical measurements		
Waist-hip ratio	**0.9 ± 0.1**	**0.90 ± 0.08**
BMI, kg/m^2^	**26 (23.6-28.6)**	**27.1 (25.0-29.7)**
Indices of arterial compliance		
Aortic distensibility, 10^-3^/mm Hg	**2.2 (1.59-2.90)**	**2.0 (1.5-2.7)**
TAC, mL/m^2^ × mm Hg	**0.7 (0.6-0.8)**	**0.6 (0.5-0.8)**
CMR indices of cardiac structure and function		
LVEDVI, mL/m^2^	**79.2 ± 13.6**	**81.5 ± 16.3**
LVESVI, mL/m^2^	**31.4 (26.5-36.9)**	**32.8 (27.4-39.3)**
LVSVI, mL/m^2^	46.9 ± 8.2	46.5 ± 8.2
LVMI, g/m^2^	**45.8 ± 8.6**	**49.0 ± 9.6**
RVEDVI, mL/m^2^	84.2 ± 15.4	83.4 ± 14.3
RVESVI, mL/m^2^	36.4 ± 9.5	36.4 ± 8.9
RVSVI, mL/m^2^	**47.7 ± 8.7**	**46.9 ± 8.5**
LVEF, %	**59.5 ± 6**	**57.7 ± 7.4**
M/V ratio, g/mL	**0.56 (0.5-0.62)**	**0.59 (0.54-0.66)**
LVGFI, %	**47.7 ± 6.7**	**45.1 ± 7.4**

Values are median (IQR) where absolute skew is ≥0.9, n (%), or mean ± SD. The data shown here are from the subjects with values that were available. **Bold** indicates differences between the 2 groups are statistically significant (*P* < 0.05).

BMI = body mass index; CMR = cardiac magnetic resonance; LVEDVI = left ventricular end-diastolic volume index; LVEF = left ventricular ejection fraction; LVESVI = left ventricular end-systolic volume index; LVGFI = left ventricle global function index; LVMI = left ventricular mass index; LVSVI = left ventricular stroke volume index; M/V = LV mass-to-volume ratio; RVEDVI = right ventricular end-diastolic volume index; RVESVI = right ventricular end-systolic volume index; RVSVI = right ventricular stroke volume index; TAC = total arterial compliance; other abbreviation as in Table 1.

### Estimated brain age in IHD

In non-IHD subjects (test set), the model showed a mean difference between the chronological age and predicted brain age (MAE) of 4.69 years (Table 3). When applying the model to the IHD group, a greater deviation of predicted brain age from actual age was observed (MAE = 6.96 years) indicating an older-appearing brain. A significant difference in mean brain-age delta values between the 2 cohorts was also found (*P <* 0.001), confirming that the IHD subjects had significantly higher brain age than those without the disease.

**Table 3:** Model Performance for Brain Age Prediction

	Non-IHD (Train)	Non-IHD (Test)	IHD
MAE, y	4.72	4.69	6.96
R^2^ (variance in age explained)	0.38	0.39	−0.76

MAE indicates the mean difference between the chronological age and the predicted brain age expressed in years, with higher values indicating older-appearing brain. R^2^ represents the proportion of variance in the predicted brain age explained by the used features in the model. In IHD group, a greater deviation of predicted brain age from chronological age was observed compared to non-IHD (test-set) (MAE = 6.96 years for IHD vs MAE = 4.69 years for non-IHD).

MAE = mean absolute error; R^2^ = coefficient of determination; other abbreviation as in Table 1.

The difference between the 2 groups was further confirmed by R^2^, with a negative value in IHD indicating that the prediction in this group was worse than the mean prediction (Supplemental Figure 3). Such negative value was mentioned in the scikit-learn documentation and reported in previous brain aging studies.^25^

The correlation value between brain-age delta and actual age in both cohorts decreased to close to zero after the estimated brain age was corrected from bias. This indicates that the correction steps were performed correctly, and the derived brain-age delta was free from age dependency. This was notably observed in the IHD cohort (Supplemental Table 6).

### Association of brain age with incident dementia

One unit increase in brain-age delta was associated with an increase of 13% in the odds of dementia occurring (odds ratio: 1.13 [95% CI: 1.04-1.22]; *P =* 0.002). Such association remained significant after accounting for imbalance between the 2 groups (dementia vs nondementia) (odds ratio: 1.15 [95% CI: 1.01-1.33]; *P =* 0.04) (Table 4).

**Table 4:** Association of Brain-Age Delta With Incident Dementia

	Dementia	Beta Value	OR (95% CI)	*P* Value
Model 1 (n = 8,389)				
	27 (0.32)	0.124	1.13 (1.04-1.22)	0.002
Model 2 (n = 54)				
	27 (50)	0.146	1.15 (1.01-1.33)	0.04

Values are n (%) or 95% CI, unless otherwise indicated. Model 1 was performed using all non-IHD (test-set) and IHD subjects. Model 2 was performed after selecting the sample using propensity score matching based on age and sex.

Abbreviation as in Table 1.

### Mediation effects of WMH on IHD and brain age

**Figure 2:**
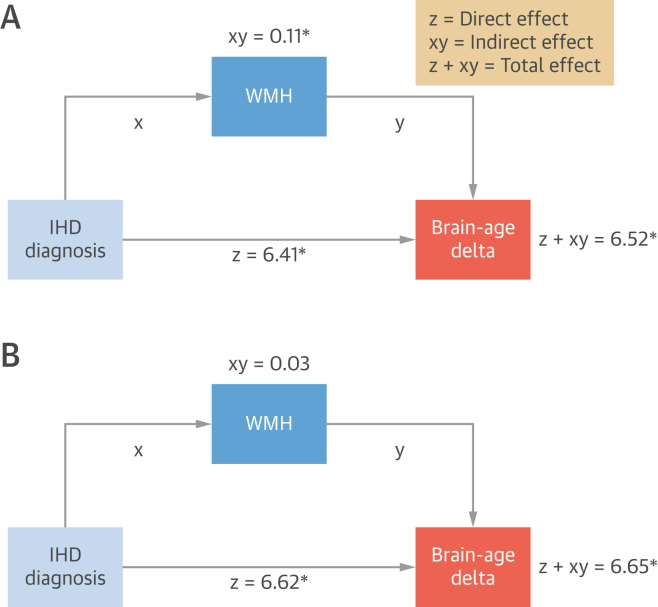
Mediation of Effects of IHD on Brain-Age Delta Unadjusted **(A)** and adjusted **(B)** models. The (beta) values indicate the effect size. An **asterisk (∗)** indicates that the association is statistically significant (*P <* 0.05). Created using BioRender.com. Abbreviations as in Figure 1.

The association of IHD diagnosis with brain-age delta can be explained as a direct effect, caused by the disease itself, an indirect effect mediated by WHM, and a total effect, which is the result of both (Figure 2).

An unadjusted model showed that IHD had significant effects on brain-age delta both directly and indirectly (*P <* 0.001). However, the direct effect of IHD on brain aging was far larger than through WMH as mediator (beta values = 6.41 vs 0.11, respectively).

When adjusted for covariates, the direct effect remained similar in size and significant (beta value = 6.62; *P <* 0.0001), while the mediation effect of WMH became nonsignificant.

### Association of brain age with vascular risk factors

Increasing brain-age delta was significantly associated with a history of diabetes in both groups. However, such effect was greater in IHD (beta values up to 2.1 years in IHD vs 1.3 years in controls).

In IHD, faster brain aging was also significantly linked with increased adiposity (larger waist-hip ratio and increased body mass index). The greatest association was found with waist-hip ratio, where for 1 unit increase of such value, there was an increase in estimated brain age of up to 8.02 years from normal aging (as indicated by the beta value) (Table 5). A similar trend was observed in control subjects, albeit the associations were not significant.

**Table 5:** Associations Between Brain-Age Delta and Vascular Risk Factors

	Non-IHD		IHD	
	Beta Value	*P* Value	Beta Value	*P* Value
Smoking^a^	0.58	0.26	0.56	1.00
Deprivation	0.05	0.33	0.05	1.00
BMI, kg/m^2^	0.03	1.00	0.1	**0.003**
Diabetes^a^	1.3	**0.001**	2.06	**<0.001**
Hypertension^a^	0.45	1.00	0.84	1.00
Hypercholesterolemia^a^	0.21	1.00	0.25	1.00
Waist-hip-ratio	1.72	0.81	8.02	**<0.001**

The beta value is interpreted as the difference in the dependent variable (brain-age delta) for changing 1 U in the independent variable (exposure). Specifically, changing 1 U in the exposure leads to increasing (positive beta value) or decreasing (negative beta value) in brain-age delta. For instance, in non-IHD, diabetes is associated with an increased brain-age delta = 1.3 years, whilst in IHD such increase is near 2.1 years. **Bold** indicates statistically significant after Bonferroni correction (*P* < 0.05).

Abbreviations as in Tables 1 and 2.

a Indicates categorical variables; the other variables are continuous.

**Figure 3:**
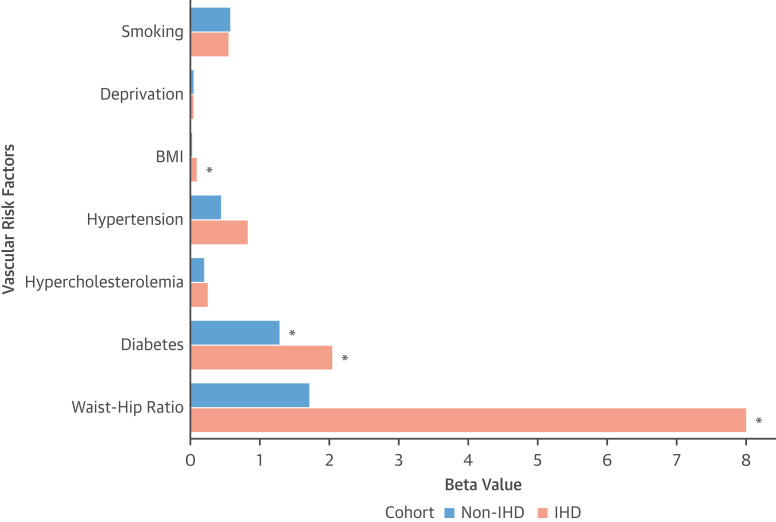
Comparing the Effect Size of Vascular Risk Factors on Brain Aging in IHD vs Non-IHD Higher beta-value (in years) indicates faster brain aging. An **asterisk (∗)** indicates that the association is statistically significant (*P <* 0.05). Created using BioRender.com. BMI = body mass index; other abbreviations as in Figure 1.

The estimated contribution of diabetes and increased adiposity on the acceleration of brain aging was overall greater in the presence of IHD (up to 10.2 years in IHD vs 3 years in controls, when summing up their beta values). The different effect size of vascular risk factors on brain aging in the 2 cohorts is shown in Figure 3.

**Central Illustration:**
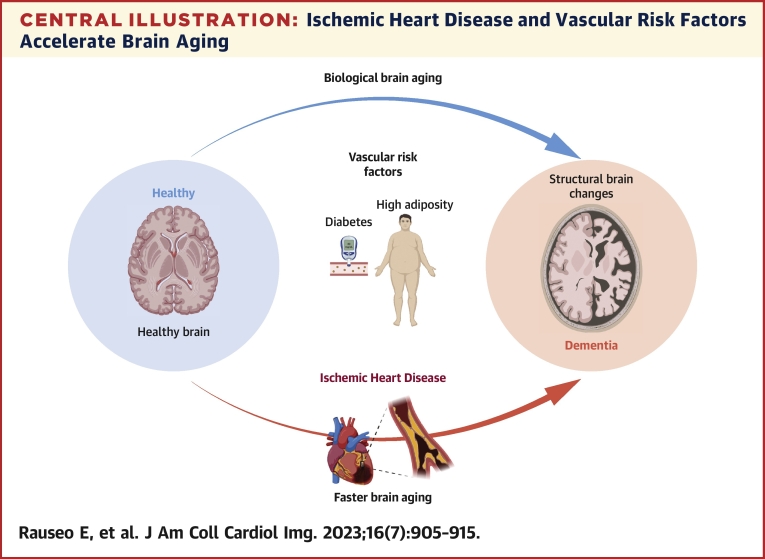
Ischemic Heart Disease and Vascular Risk Factors Accelerate Brain Aging Brain aging estimated from magnetic resonance imaging data, reflects structural brain changes. Ischemic heart disease and coexisting vascular risk factors can accelerate biological brain aging and increase the risk of dementia. Created using BioRender.com.

### Association of brain age with CMR parameters

In non-IHD subjects, increasing brain-age delta was significantly associated with smaller LV and RV volumes, and lower indices of cardiac function, LV mass, aortic distensibility, and TAC values. Similar direction of associations was found in the IHD cohort for all imaging metrics, albeit not statistically significant (Table 6). None of the CMR metrics were significantly associated with the risk of dementia (Supplemental Table 7).

**Table 6:** Associations Between Brain-Age Delta and Cardiovascular Imaging Parameters

	Non-IHD		IHD	
	Beta Value	*P* Value	Beta Value	*P* Value
LVEDVI, mL/m^2^	−0.03	**<0.001**	−0.01	0.65
LVEF, %	−0.03	0.05	0.01	1.00
LVESVI, mL/m^2^	−0.03	**0.001**	−0.02	0.82
LVMI, g/m^2^	−0.03	**<0.001**	−0.004	1.00
LVSVI, mL/m^2^	−0.05	**<0.001**	−0.01	1.00
RVEDVI, mL/m^2^	−0.03	**<0.001**	−0.02	0.27
RVESVI, mL/m^2^	−0.03	**<0.001**	−0.03	0.95
RVSVI, mL/m^2^	−0.05	**<0.001**	−0.03	0.86
LVGFI, %	−0.04	**<0.001**	0.001	1.00
M/V ratio, g/mL	1.41	0.75	2.19	1.00
Aortic distensibility, 10^−3^/mm Hg	−0.37	**<0.001**	−0.07	1.00
TAC, mL/m^2^ × mm Hg	−1.65	**<0.001**	−1.29	0.66

The beta value is interpreted as the difference in the dependent variable (brain-age delta) for changing 1 U in the independent variable (exposure). Specifically, changing 1 U in the exposure leads to increasing (positive beta value) or decreasing (negative beta value) in brain-age delta. **Bold** indicates statistically significant after Bonferroni correction (*P* < 0.05).

Abbreviations as in Tables 1 and 2.

## Discussion

In this study, we estimated the brain age of IHD subjects from the UKB using volumetric brain features extracted from MRI data. We found that subjects with IHD showed, on average, a more advanced brain age than their chronological age, with their brains appearing significantly older than those without the disease. Accelerated brain aging was also linked with increased risk of dementia. WMH, a marker of cerebrovascular injury, did not significantly mediate the effects of IHD on brain aging, suggesting that other mechanisms related to the disease itself were involved in the association. IHD, diabetes, and increased adiposity were associated with even faster brain aging. Overall, these findings suggest that IHD-related mechanisms and vascular risk factors concur to accelerate biological brain aging and so the risk of developing dementia (Central Illustration).

### IHD and brain health

Although our results cannot be directly compared with previous studies given the different approaches used to evaluate the impact on brain structures, they are consistent with existing research and confirm the association between IHD and poorer brain health.^4^ There is evidence to support faster cognitive deterioration in the years following the diagnosis of IHD than before, suggesting possible disease-related mechanisms implicated in the association.^26^

We observed that IHD subjects had early structural brain changes in the form of brain atrophy and increased WMHs than their peers. WMHs reflect underlying cerebral small vessel disease, a form of vascular dysfunction which may occur both in the brain and the heart, leading to different clinical manifestations, including ischemia, cognitive dysfunction, and dementia.^27^^,^^28^ However, we observed that the vascular contributions to brain aging in IHD, as expressed by WMHs, were nearly insignificant. Instead, it was mainly a direct effect of the disease to drive the association. These results confirm the strong association between IHD and brain deterioration, and that such relationship is not fully explained by WMHs.^6^^,^^7^

Our findings suggest that IHD contributes to brain aging through several disease-related mechanisms that this marker of cerebrovascular injury could not capture. Among the mechanisms involved are direct ischemic brain injury, blood-brain barrier alterations, and various biological pathways, including oxidative stress, immune responses, and endothelial dysfunction.^5^ Future studies using more sensitive measures of brain microstructural changes are needed to establish the exact pathophysiological pathways linking IHD, brain aging, and cognitive decline in the long term.

### The role of coexisting vascular risk factors in brain aging

Vascular risk factors can augment the risk of dementia both through an increased risk of cardiac and cerebrovascular diseases and as independent risk factors.^2^ These potentially modifiable conditions promote systemic atherosclerosis which may directly affect brain and cardiovascular health.^6^ When IHD and vascular risk factors coexist, the negative effects on cerebral autoregulation and brain perfusion might be further enhanced thus resulting in accelerating brain aging. In this view, concomitant IHD may be seen as a more advanced, symptomatic end-organ vascular disease and as a surrogate marker of clinically significant atherosclerosis affecting the brain.^7^

Similarly, we observed that increased adiposity and diabetes were associated with significantly faster brain aging in presence of IHD. This indicates that the impact of such factors on brain structures is amplified when there is a coexisting diagnosis of IHD with a synergistic effect of vascular and ischemic-related processes on disease progression.

Diabetes played a significant role in brain aging even in the absence of IHD, confirming that this condition is one of the strongest risk factors for dementia.^29^ We also found a very strong association between indices of adiposity and brain aging in IHD. A higher waist-to-hip ratio, a measure of abdominal obesity, was associated with the greatest increase in brain aging. This suggests that central adiposity as an indicator of visceral fat might be the component that mainly contributes to accelerating brain aging in the presence of IHD. Visceral adipose tissue inducing insulin resistance and increased systemic inflammation might play a key role in causing vascular endothelial dysfunction and eventually subclinical brain damage.^30^ Thus, we hypothesize that coexisting IHD and increased central adiposity might represent a condition of higher vascular risk burden. In this view, several factors might interact and lead to a greater risk of structural and vascular cerebral changes potentially resulting in more accelerated brain aging.

### The role of cardiovascular hemodynamics in brain aging

In the absence of IHD, we found significant associations between accelerated brain aging and cardiovascular changes typically observed in aging hearts: smaller ventricular volumes, reduced LV global performance, and poorer aortic compliance. However, such alterations did not translate into an increased risk of dementia. These changes in cardiovascular hemodynamics likely reflect the coupling of ventricular and vascular stiffening processes over the lifetime, which can occur even in the absence of explicit cardiovascular conditions, with a potential impact on brain health before the onset of cognitive impairment.^14^^,^^31^

None of the CMR measures analyzed was significantly linked with brain aging in IHD. Although that can be due to the smaller number of IHD subjects, other types of cardiac changes, likely ischemia-related and not captured by conventional indices of cardiac function and structure, might play a role in the association with brain aging. Further studies using advanced imaging metrics and integrating a more comprehensive assessment of the extent of myocardial ischemia are thus needed to shed light on the complex relationship between IHD and brain aging.^32^

### Clinical implications

Our findings highlight the importance of preventing IHD and promoting healthier behaviors to delay brain deterioration and possible dementia. Brain age can be considered an attractive communication tool to inform patients about their risk status and the toll of vascular risk factors on the brain’s health. Communicating the risk in age has been shown to increase risk perceptions and to provide greater emotional impact, such as further motivating patients to make lifestyle changes.^12^ That can be particularly useful for younger individuals who may perceive their absolute short-term risk as too low to be emotionally impactful due to their young age.^33^ Emotional reactions can play a role in rational behavior by influencing the perception of risk in a more intuitive, rapid, and understandable way than cognitive evaluations.^34^ However, future prospective trials showing the effectiveness of using brain age for communicating risk information are needed before recommending such an approach in clinical practice.

### Study limitations

We used volumetric brain features to estimate brain age as they were previously identified among the top informative predictors. However, including multiple imaging modalities could lead to better model performance.

The number and type of brain IDPs used in the model can affect the estimated value of brain-age delta. However, our purpose was to use this parameter to show the differences in brain aging rate between IHD vs non-IHD rather than propose a quantification of aging itself.

Additional factors, including genetics and early brain developments, can likely influence brain structures. Future studies based on longitudinal imaging data could help determine such factors' role in the individual change trajectories of brain aging.

The average actual age of IHD and non-IHD differed significantly. However, we applied an age-bias correction method that eliminated the effects of age from the estimated brain age delta.

We assessed the effect of certain previously validated exposures linked to brain health. Additional covariates, including biological, psychological, and behavioral parameters, were not evaluated. That was done to avoid model overfitting, potentially decreasing the sensitivity to real effects. Differences in sample size, age range, and methods used to select covariates and brain features might explain some discrepancies between the current and previous brain aging results on UKB.

Although the direction of associations between exposures and brain-age delta might indicate the potential impact of the exposure itself on brain aging (accelerating vs decelerating), these results do not allow for causal inference. We cannot infer temporal relationships between brain aging and exposures as this is a cross-sectional study. Future longitudinal investigations using serial brain and CMR data linked with clinical outcomes might evaluate the effect of having an older appearing brain in the long term.

## Conclusions

Prevalent IHD is associated with accelerated brain aging and increased risk of dementia, that is not fully explained by microvascular injury. Besides shared vascular risk factors, additional disease-related mechanisms might contribute to abnormal aging. Using brain age to communicate the risk levels for cognitive deterioration may increase patients’ awareness and improve their adherence to therapies and lifestyle changes.

Perspectives **COMPETENCY IN MEDICAL KNOWLEDGE:** Our findings highlight the importance of looking for early signs of poorer brain health in the form of subtle (volumetric) changes in brain structures in subjects with prevalent IHD and vascular risk factors.

**TRANSLATIONAL OUTLOOK 1:** Brain age can help identify IHD subjects at higher risk of developing cognitive deterioration who may benefit from early and more aggressive treatment and preventative interventions.

**TRANSLATIONAL OUTLOOK 2:** Brain age can be used to efficiently communicate the risk of brain deterioration to patients and promote healthy behaviors.

**TRANSLATIONAL OUTLOOK 3:** Given the projected increase of prevalent IHD and dementia due to global population aging, there is a need for insights into causal mechanisms underlying heart-brain interactions and early signs of cognitive impairment before overt symptoms.

## Funding Support and Author Disclosures

Barts Charity (G-002346) contributed to fees required to access UK Biobank data (access application #2964). Drs Rauseo and Petersen have received grants from the London Medical Imaging and Artificial Intelligence Centre for Value Based Healthcare (AI4VBH), which is funded from the Data to Early Diagnosis and Precision Medicine strand of the government’s Industrial Strategy Challenge Fund, managed and delivered by Innovate UK on behalf of UK Research and Innovation (UKRI). Mr Salih is supported by INVITE program co-financed by the European Union within the Horizon 2020 Programme and by the Regione del Veneto. Dr Raisi-Estebragh has received grants from the National Institute for Health Research (NIHR) Integrated Academic Training programme which supports her Academic Clinical Lectureship post; she has also received grants from the British Heart Foundation Clinical Research Training Fellowship (No. FS/17/81/33318). Dr Aung has received grants from the NIHR Integrated Academic Training programme which supports his Academic Clinical Lectureship post, and the Academy of Medical Sciences Starter Grant for Clinical Lecturers (SGL024∖1024). Dr Slabaugh has received consulting fees from MindRank AI. Dr Marshall has received grants from Barts Charity; and has received personal fees from Biogen and GE Healthcare. Dr Neubauer has received grants from the Oxford NIHR Biomedical Research Centre. Drs Galazzo and Menegaz have received grants from the Fondazione CariVerona (EDIPO project—reference number 2018.0855.2019). Dr Petersen has received grants from the NIHR Biomedical Research Centre at Barts, and from the British Heart Foundation for funding the manual analysis to create a cardiovascular magnetic resonance imaging reference standard for the UK Biobank imaging resource in 5,000 CMR scans (www.bhf.org.uk; PG/14/89/31194), and from the SmartHeart EPSRC programme grant (www.nihr.ac.uk; EP/P001009/1); and has received consulting fees from Circle Cardiovascular Imaging Inc, Calgary, Alberta, Canada. All other authors have reported that they have no relationships relevant to the contents of this paper to disclose.
