# Supplementary material for: Ischemic Heart Disease and Vascular Risk Factors Are Associated With Accelerated Brain Aging
Source: JACC Cardiovasc Imaging. 2023 Jul;16(7):905–15. doi: 10.1016/j.jcmg.2023.01.016 (PMC10317841; doi:10.1016/j.jcmg.2023.01.016)
Supplement: Supplemental Data [file mmc1.docx]

**Supplemental Methods**

**Brain MRI feature extraction**

We used imaging-derived phenotypes (IDP)s resulted from an image pre-processing pipeline developed by the UK Biobank (UKB) working group described in details elsewhere (1). Further protocols details are available online at: <https://biobank.ctsu.ox.ac.uk/crystal/crystal/docs/brain_mri.pdf>.

In brief, pre-processing structural MRI included face removal, brain extraction, linear alignment to standard MNI152 brain template. Brain MRIs were segmented into three categories that are: white matter, grey matter, and cerebrospinal fluid using a Functional MRI of the Brain (FMRIB)’s Automated Segmentation Tool (FAST). Then, the segmented data were used to perform a SIENAX-style analysis (Structural Image Evaluation, using Normalization, of Atrophy: Cross-sectional) (2). Volumes of different regions were calculated both normalized and not normalized to head size to generate the IDPs accessible through the UKB showcase. The subcortical structural volumes for each hemisphere were calculated using, an FMRIB’s Integrated Registration and Segmentation tool (FIRST). The total volume of white matter hyperintensities (WMH), used in the mediation analysis, was calculated using T2-weighted brain MRI data and the lesion is segmented using the Brain Intensity Abnormality Classification Algorithm (BIANCA) tool.

**Statistical analysis**

To assess whether IHD was associated with faster brain aging, whether it, in turn, was related to the risk of dementia, and to investigate potential mechanistic links between IHD and brain aging, including the role of some risk factors, we used a four-staged approach, which can be summarized as follows (**Supplemental Figure 1**): A) estimating brain age in IHD; B) assessing the relationship between brain age and risk of dementia; C) evaluating the role of WMH as a potential mediator in the association between IHD and brain aging; D) evaluating the association of brain aging with vascular risk factors and imaging parameters. A detailed description of each stage of our analysis is provided below.

Differences between groups were assessed using proportions t-tests for categorical variables and Student’s t-tests with unequal variances for continuous variables, with p < 0.05 considered statistically significant for all tests (corrected for multiple comparisons).

*Brain age estimation*

Brain age was estimated using Python 3.8.10 and Scikit-learn version 0.23.2. Bayesian Ridge regression was used as a regression model to estimate brain age as it was shown to provide competitive performance (3). In the model, the 25 brain MRI features (described in **Supplemental Table 2**) were the independent variables while the chronological age was the dependent variable. The features were normalized to zero mean and unit variance to account for different measurement scales. The actual age was demeaned (shifted to have zero mean) before fitting it into the model to have a centered version of the outcome (4). Sex, education level, height, and volumetric scaling from T1 head image to standard space were used as confounds as they can significantly affect the outcome. The confounds were regressed from the features using a linear regression model prior to modeling brain age.

The brain aging model was built based on participants with non-IHD by splitting the data into two subsets (training set, 80%; testing set, 20%). The model performance for both training and test datasets was assessed using the Mean Absolute Error (MAE) and the coefficient of determination (R2). MAE in brain age studies is interpreted as the deviation between predicted brain age and the chronological age expressed in years, with higher values indicating older appearing brains. R2 represents the proportion of variance in the predicted brain age explained by the used features in the model.

Brain age estimation may involve an observed bias, with age underestimation in older participants, overestimation in younger subjects and more accurate estimation for those with ages close to the mean age (5). We removed the age-dependency bias using the statistical method described previously (6)(3). In brief, we calculated the slope (α) and the intercept (β) from the training data as follow: D= α*Ω+β, where D is the brain age delta and Ω is the actual age. Then we used the slope and the intercept to correct the estimated brain age in the test dataset as follows: corrected predicted brain age = predicted brain age – (α*Ω+β). After bias-correction, we calculated brain-age delta by subtracting the chronological age from the predicted brain age in the test datasets, with positive values (expressed in years) indicating accelerated brain aging. Pearson correlation was also calculated between actual age and brain-age delta, before and after bias correction.

Next, brain age was estimated on IHD subjects using the previously trained model to assess the deviation of the brain-age delta from the reference (non-IHD) population. The same steps of features preparation and bias correction using the parameters calculated on the training data were applied. IHD and non-IHD groups were compared in terms of model performance and difference in mean brain-age delta, the latter considered as a measure of apparent brain aging (4).

*Relationship between brain age and risk of dementia*

To evaluate whether brain age was related with risk of dementia, we studied the association between brain-age delta and incident dementia using logistic regression on a sample comprising both IHD and non-IHD (test set) subjects (n = 8,389). Among them, 27 developed dementia after the imaging visit (incident event). Criteria used to define dementia based on selected UKB fields are reported in **Supplemental Table 6**.

Logistic regression model was fitted using brain-age delta as the predictor variable and incident dementia (0 = non-dementia, 1= dementia) as the response variable. The model was adjusted for age, sex, and education level.

Since there were fewer subjects with incident dementia than those without (27 vs 8,362, respectively), to account for imbalance between the two groups, we used propensity score matching based on age and sex to identify an equal number of non-dementia subjects. We then repeated the association analysis using logistic regression on the 54 subjects (dementia, n=27 vs non-dementia, n=27) adjusted for education level. The association between brain-age delta and risk of dementia are presented in terms of beta value, odds ratio (OR) and 95% confidence interval (CI).

*Mediation effects of WMH on IHD and brain age*

We performed a mediation analysis to test to what extent the effects of IHD on brain aging (as expressed by brain-age delta) were mediated by WMH, a proxy of cerebrovascular injury. The analysis was conducted using ordinary least squares regression, as described in a previous publication (7). In the model, the input was whether the subject had IHD or not (0, 1), the output was the brain-age delta, and the mediator was WMH. For this analysis, the associations between variables were described using the term effect as per statistical convention. Specifically, we evaluated the following associations' pathways: 1) IHD with brain-age delta (direct effect), indicating that IHD directly affects brain aging without any mediator; 2) IHD with brain-age delta through WMH (indirect effect), indicating that the effects of IHD on brain aging are indirect as WMH mediates them. The total effect indicates the combination of both direct and indirect effects of IHD on brain aging.

We conducted both unadjusted and adjusted analyses using sex, education level, age, height, and volumetric scaling from the T1 head image to standard space as covariates. We used the tool PROCESS (R & SPSS) as an implementation of the mediation analysis.

*Association of brain age with vascular risk factors and imaging parameters*

To assess the role of vascular risk factors and imaging parameters to model brain age, the association between brain-age delta values and such exposures was evaluated using a linear regression model in both IHD and non-IHD (test-set) groups. In the model, the brain-age delta was the dependent variable while the exposure was the independent variable adjusted for the same confounds as before plus age. The p-values were corrected for multiple comparisons using the Bonferroni method (alpha= 0.05; number of tests = 19). In the regression model we used unstandardized measures to reveal the effect (beta value) of changing one unit in the exposure on the brain-age delta. Specifically, changing the exposures may lead to increasing or decreasing in brain-age delta (estimated in years) based on the direction of the association (positive vs negative beta value).

Finally, to evaluate whether the imaging parameters were actually linked with the risk of dementia, we studied the association between the CMR metrics and incident dementia using logistic regression. This test was conducted only on the non-IHD group (test-set) (n= 8,389) as this was the only subset where we observed significant associations between brain-age delta and some imaging parameters. The number of non-IHD (test-set) subjects with available CMR metrics who developed dementia (incident event) was 8. Logistic regression model was fitted using the CMR measures as the predictor variable and incident dementia (0 = non-dementia, 1= dementia) as the response variable. The model was adjusted for age and sex.

**Supplemental References**

1. Miller KL., Alfaro-Almagro F., Bangerter NK., et al. Multimodal population brain imaging in the UK Biobank prospective epidemiological study. Nat Neurosci 2016;19(11):1523–36. Doi: 10.1038/nn.4393.

2. Smith SM., Zhang Y., Jenkinson M., et al. Accurate, robust, and automated longitudinal and cross-sectional brain change analysis. Neuroimage 2002;17(1):479–89. Doi: 10.1006/nimg.2002.1040.

3. Salih A., Galazzo IB., Jaggi A., et al. Multi-modal Brain Age Estimation: A Comparative Study Confirms the Importance of Microstructure. Math Vis 2021:239–50. Doi: 10.1007/978-3-030-73018-5_19.

4. Smith SM., Vidaurre D., Alfaro-Almagro F., Nichols TE., Miller KL. Estimation of brain age delta from brain imaging. Neuroimage 2019;200(February):528–39. Doi: 10.1016/j.neuroimage.2019.06.017.

5. de Lange AMG., Cole JH. Commentary: Correction procedures in brain-age prediction. NeuroImage Clin 2020;26. Doi: 10.1016/j.nicl.2020.102229.

6. Beheshti I., Nugent S., Potvin O., Duchesne S. Bias-adjustment in neuroimaging-based brain age frameworks: A robust scheme. NeuroImage Clin 2019;24:102063. Doi: 10.1016/j.nicl.2019.102063.

7. Hayes Andrew F. Introduction to Mediation, Moderation, and Conditional Process Analysis - Model Numbers. vol. 46. 2013.

**Supplemental Figure 1. Illustration of the staged approach**


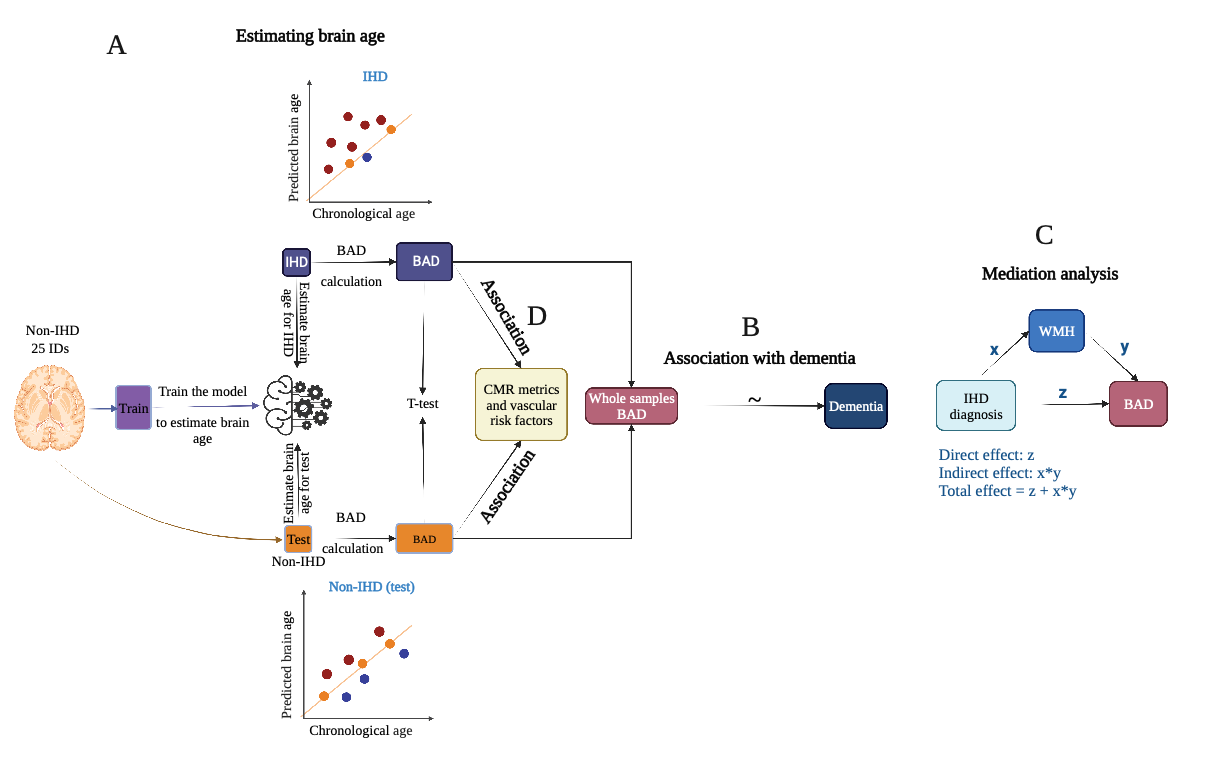


**A** – Brain age and its deviation from chronological age (brain-age delta, a marker of brain aging), was estimated in subjects with prevalent IHD using a Bayesian ridge regression model with 25 structural (volumetric) brain MRI features (IDPs) and built using UK Biobank participants with non-IHD. **B** – Validation of brain aging model by studying its association with dementia risk using logistic regression analysis. **C** – Evaluating potential mechanistic links between IHD and brain aging using mediation analysis: to what extent WMH, marker of microvascular injury, mediated the effects of IHD on brain aging. **D** – Evaluating the association of vascular risk factors and CMR metrics with brain aging in IHD vs non-IHD using linear regression analysis. IHD, ischemic heart disease; BAD, brain-age delta; IDPs, imaging-derived phenotypes; WMH, white matter hyperintensity.

**Supplemental Figure 2. Distribution of actual age range in IHD vs non-IHD groups**


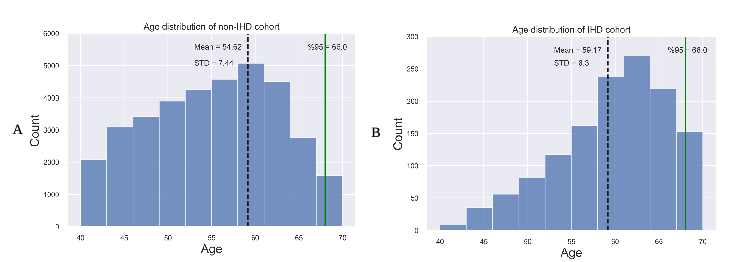


The two cohorts showed a similar age range (40-70), albeit the distribution was slightly skewed to the left in the IHD cohort. The black dashed line indicates the mean value; the solid green line indicates the 95% percentile. The mean age for non-IHD (A) was 54.6 ± 7.4 years, and for IHD (B) was 59.2 ± 6.3 years. Very few participants had an age range above the 95% percentile in both groups.

**Supplemental Figure 3. Predicted brain age in both groups**


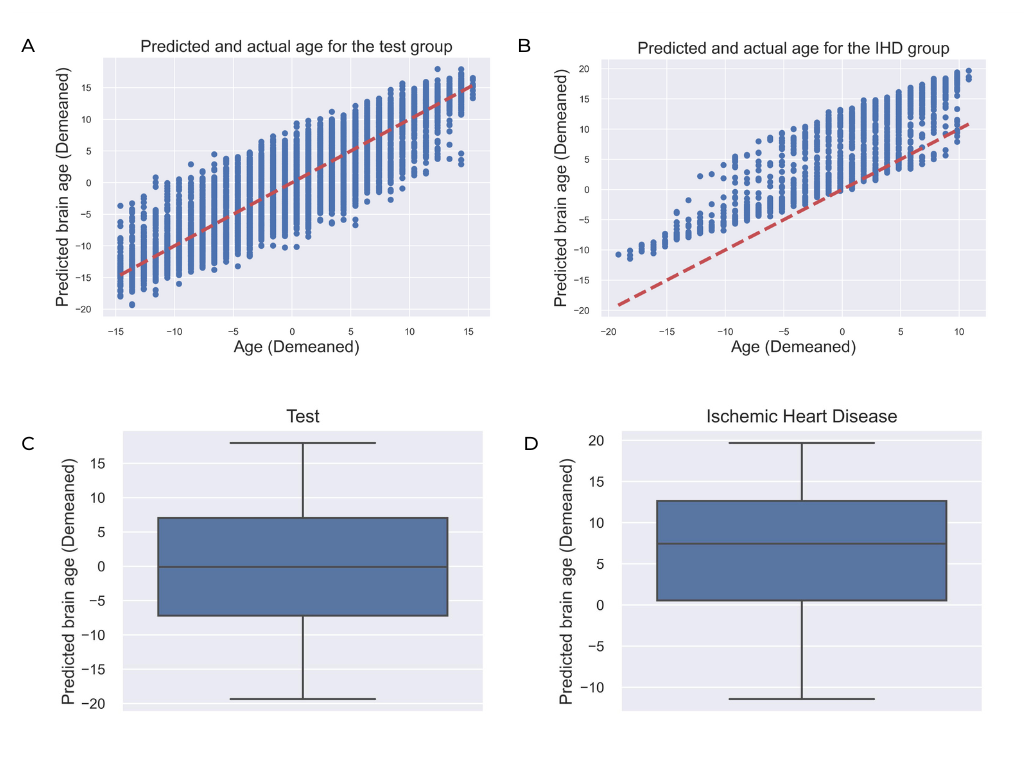
The regression line (red dashed) from the trained model fits well with the test (non-IHD) (A) but not with the IHD data (B), explaining the higher MAE value in the IHD group. The age ranges in both cohorts appear different from the actual ones (40-70) because age was demeaned before fitting into the model. The boxplots (C, D) describe the distribution of predicted brain age (demeaned) in both cohorts, with the mean (median) value, and the first and third quartiles. The mean predicted brain age for IHD (D) is higher than for the test group (C). Furthermore, both cohorts have no predicted brain age as an outlier.

**Supplemental Table 1. List of ICD-10 codes used to define ischemic heart disease from the UK Biobank showcase**

| **Source** | **UKB Field** | **ICD-10 value** | **Description** |
| --- | --- | --- | --- |
| ICD10 Summary diagnoses | 41270, 41280 | I20 | Angina pectoris |
|  |  | I21 | Acute myocardial infarction |
|  |  | I22 | Subsequent myocardial infarction |
|  |  | I23 | Certain current complications following acute myocardial infarction |
|  |  | I24 | Other acute ischaemic heart diseases |
|  |  | I25 | Chronic ischaemic heart disease |

**Supplemental Table 2. UK Biobank data sources for ascertainment of brain imaging-derived phenotypes (IDP)s**

| **UKB Field** | **Description** |
| --- | --- |
| **25 brain IDPs used in the brain age model** | |
| ***T1 structural brain MRI*** |  |
| 25001 | Volume of peripheral cortical grey matter (normalised for head size) |
| 25002 | Volume of peripheral cortical grey matter |
| 25003 | Volume of ventricular cerebrospinal fluid (normalised for head size) |
| 25004 | Volume of ventricular cerebrospinal fluid |
| 25005 | Volume of grey matter (normalised for head size) |
| 25006 | Volume of grey matter |
| 25007 | Volume of white matter (normalised for head size) |
| 25008 | Volume of white matter |
| 25009 | Volume of brain, grey + white matter (normalised for head size) |
| 25010 | Volume of brain, grey + white matter |
| 25025 | Volume of brain stem + 4th ventricle |
| ***Subcortical volumes (FIRST)*** |  |
| 25011 | Volume of thalamus (left) |
| 25012 | Volume of thalamus (right) |
| 25013 | Volume of caudate (left) |
| 25014 | Volume of caudate (right) |
| 25015 | Volume of putamen (left) |
| 25016 | Volume of putamen (right) |
| 25017 | Volume of pallidum (left) |
| 25018 | Volume of pallidum (right) |
| 25019 | Volume of hippocampus (left) |
| 25020 | Volume of hippocampus (right) |
| 25021 | Volume of amygdala (left) |
| 25022 | Volume of amygdala (right) |
| 25023 | Volume of accumbens (left) |
| 25024 | Volume of accumbens (right) |
| **Brain IDP used in the mediation analysis** | |
| ***T2-weighted brain MRI*** |  |
| 25781 | Total volume of white matter hyperintensities (from T1 and T2_FLAIR images) |

**Supplemental Table 3. UK Biobank data sources for ascertainment of clinical exposures**

| **Source** | **UKB Field** | **Description** |
| --- | --- | --- |
| ***Body Mass Index (BMI)*** |  |  |
| Body size measures: Standing height, weight | 50, 21002 | BMI (Kg/m^2^) = weight (kg)/ height^2^ (m) |
| ***Diabetes*** |  |  |
| Diagnosed by doctor | 2443 | Diabetes |
| Self-reported medication | 6177, 6153 | Insulin |
| Blood biochemistry | 30750 | Glycated haemoglobin (HbA1c) > 48 mmol/mol |
| ***Hypercholesterolemia*** | |  |
| Self-reported medication | 6177, 6153 | Cholesterol lowering medication |
| Blood biochemistry | 30690 | Serum total cholesterol >7 mmol/L |
| ***Hypertension*** |  |  |
| Self-reported medication | 6177, 6153 | Blood pressure medication |
| ***Smoking (current smoker)*** |  |  |
| Self-report | 1239 | Yes, on most or all days  Only occasionally |
| ***Townsend index*** |  |  |
| Baseline characteristics | 189 | Townsend deprivation index at recruitment |
| ***Waist-hip-ratio*** |  |  |
| Body size measures:  Waist circumference, hip circumference | 48,49 | Waist-hip-ratio = Waist circumference/ Hip circumference |

**Supplemental Table 4. UK Biobank data sources for ascertainment of incident dementia**

| **Data source** | **Data-Field** | **Code** |
| --- | --- | --- |
| Non-cancer illness code, self-reported | 20002 | 1263 |
| ICD10 | 41270 | A810, F106, G300, G301, G308  F000, F001, F002, F009, G310  G311, G318, F010, F011, F012  F013, F018, F019, F020, F021  F022, F023, F024, F028, F03, F051 |
| ICD9 | 41271 | 2900, 2904, 2941, 3310, 3312, 3315 |
| First occurrence | 130836 | Date F00 first reported (dementia in Alzheimer’s disease) |
|  | 130838 | Date F01 first reported (vascular dementia) |
|  | 130840 | Date F02 first reported (dementia in other diseases classified elsewhere) |
|  | 130842 | Date F03 first reported (unspecified dementia) |
|  | 131036 | Date G30 first reported (Alzheimer’s disease) |

**Supplemental Table 5. Volumes of brain structures (IDPs) in non-IHD vs IHD cohorts**

| **Brain IDPs** | **Non-IHD cohort** | | **IHD cohort** | | **P-value** |
| --- | --- | --- | --- | --- | --- |
|  | **mean** | **std** | **mean** | **std** |  |
| **Volume of peripheral cortical grey matter (normalised for head size)** | 618959.8 | 40626.84 | 599087.3 | 40014.86 | < 0.001 |
| **Volume of peripheral cortical grey matter** | 480148 | 46281.87 | 473869.2 | 44952.75 | < 0.001 |
| **Volume of ventricular cerebrospinal fluid (normalised for head size)** | 46237.34 | 19787.14 | 53153.54 | 21182.61 | < 0.001 |
| **Volume of ventricular cerebrospinal fluid** | 36319.79 | 17036.72 | 42494.42 | 18274.66 | < 0.001 |
| **Volume of grey matter (normalised for head size)** | 794023.4 | 47572.36 | 768429.7 | 47479.02 | < 0.001 |
| **Volume of grey matter** | 615842.1 | 55827.31 | 607745.1 | 54850.85 | < 0.001 |
| **Volume of white matter (normalised for head size)** | 702272.1 | 40667.01 | 698004 | 41216.11 | < 0.001 |
| **Volume of white matter** | 545987.3 | 61865.18 | 553256.1 | 60999.43 | < 0.001 |
| **Volume of brain, grey + white matter (normalised for head size)** | 1496296 | 72794.98 | 1466434 | 70042.4 | < 0.001 |
| **Volume of brain, grey + white matter** | 1161829 | 111840 | 1161001 | 108259.5 | 0.790 |
| **Volume of thalamus (left)** | 7763.514 | 770.7964 | 7652.83 | 760.4853 | < 0.001 |
| **Volume of thalamus (right)** | 7570.728 | 746.9204 | 7467.788 | 742.5225 | < 0.001 |
| **Volume of caudate (left)** | 3381.543 | 425.1762 | 3392.709 | 426.7801 | 0.350 |
| **Volume of caudate (right)** | 3564.397 | 448.1929 | 3579.341 | 451.284 | 0.244 |
| **Volume of putamen (left)** | 4771.879 | 605.4202 | 4738.842 | 601.0369 | 0.057 |
| **Volume of putamen (right)** | 4828.078 | 595.7929 | 4800.423 | 598.7282 | 0.105 |
| **Volume of pallidum (left)** | 1757.298 | 246.4241 | 1756.316 | 257.1644 | 0.889 |
| **Volume of pallidum (right)** | 1802.18 | 246.7956 | 1784.172 | 257.2562 | 0.011 |
| **Volume of hippocampus (left)** | 3785.05 | 482.4387 | 3723.634 | 499.445 | < 0.001 |
| **Volume of hippocampus (right)** | 3898.62 | 494.3978 | 3833.532 | 527.2664 | < 0.001 |
| **Volume of amygdala (left)** | 1263.909 | 247.5637 | 1287.664 | 257.5384 | 0.001 |
| **Volume of amygdala (right)** | 1226.507 | 273.073 | 1249.522 | 279.9848 | 0.003 |
| **Volume of accumbens (left)** | 494.9743 | 120.8047 | 477.5845 | 126.1691 | < 0.001 |
| **Volume of accumbens (right)** | 389.2074 | 111.4514 | 365.6991 | 112.4412 | < 0.001 |
| **Volume of brain stem + 4th ventricle** | 22885.63 | 2889.264 | 22917.28 | 2888.373 | 0.702 |
| **Total volume of white matter hyperintensities (from T1 and T2_FLAIR images)** | 4858.15 | 6323.31 | 6724 | 7612.60 | < 0.001 |

Values (expressed in mm^3^) are presented as mean ± standard deviation with the p-value for the difference (assessed with the t-test) between the two cohorts. IHD = ischemic heart disease.

**Supplemental Table 6. Correlation between brain-age delta and actual age before and after age-bias adjustment**

| **Correlations** | **Non-IHD**  **Train** | **Non-IHD**  **Test** | **IHD** |
| --- | --- | --- | --- |
| Correlation between predicted age and chronological age before correction | 0.62 | 0.62 | 0.55 |
| Correlation between brain age-delta and chronological age before correction | -0.001 | -0.001 | -0.70 |
| Correlation between predicted age and chronological age after correction |  | 089 | 0.85 |
| Correlation between brain age-delta delta and chronological age after correction |  | 0.004 | 0.02 |

The correlation value between brain-age delta and chronological age in both cohorts decreased to close to zero after the predicted brain age was corrected from bias, indicating that the derived brain-age delta was free from age dependency.

**Supplemental Table 7: Association of CMR indices with incident dementia in non-IHD**

| **Feature** | **Beta value** | **P value** |
| --- | --- | --- |
| LVEF, % | -0.0002 | 0.07 |
| LVEDVI, ml/m2 | 0.0000 | 1 |
| LVESVI, ml/m2 | 0.0000 | 1 |
| LVSVI, ml/m2 | -0.0001 | 1 |
| LVMI, g/m2 | 0.0000 | 1 |
| RVEDVI, ml/m2 | -0.0001 | 1 |
| RVESVI, ml/m2 | 0.0000 | 1 |
| RVSVI, ml/m2 | -0.0001 | 0.45 |
| TAC, ml/m2 x mmHg | -0.002 | 1 |
| M/V ratio, g/ml | -0.003 | 1 |
| Aortic distensibility, 10^-3^/mmHg | -0.001 | 1 |
| LVGFI, % | -0.0002 | 0.05 |

LVEDVI = left ventricular end-diastolic volume index, LVEF = left ventricular ejection fraction, LVESVI = left ventricular end-systolic volume index, LVMI = left ventricular mass index, LVSVI = left ventricular stroke volume index, RVEDVI = right ventricular end-diastolic volume index, RVESVI = right ventricular end-systolic volume index, RVSVI = right ventricular stroke volume index, LVGFI = left ventricle global function index, M/V = LV mass-to-volume ratio, TAC = total arterial compliance.
